# Clinical Outcomes of Next-Generation Microwave Thermosphere Ablation for Hepatocellular Carcinoma with Primarily Hepatitis-Related Etiology

**DOI:** 10.3390/jcm12247577

**Published:** 2023-12-08

**Authors:** Shinichiro Nakamura, Toshifumi Tada, Masahiko Sue, Yu Matsuo, Shiho Murakami, Toshiro Muramatsu, Kazuhiko Morii, Hiroyuki Okada

**Affiliations:** Department of Internal Medicine, Japanese Red Cross Society Himeji Hospital, 1-12-1 Shimoteno, Himeji 670-8540, Japan; tadat0627@gmail.com (T.T.); yqxrw014@gmail.com (M.S.); moriikazuhiko@gmail.com (K.M.); h-okada@himeji.jrc.or.jp (H.O.)

**Keywords:** microwave thermosphere ablation, hepatocellular carcinoma, recurrence, safety

## Abstract

Background and aim: We investigated the clinical outcomes of patients with hepatocellular carcinoma (HCC) who underwent next-generation microwave thermosphere ablation (MTA). Methods: A total of 429 patients with 607 HCCs (maximum tumor diameter ≤40 mm) were included. We defined the following areas of the liver as those where MTA therapy is difficult to perform: caudate lobe and areas near the primary and secondary branches of the intrahepatic portal vein, inferior vena cava, gallbladder, heart, duodenum, abdominal esophagus, collateral veins around the liver, and spleen. Factors which predisposed patients to local tumor recurrence in the context of tumor location and complications were examined. Results: The primary etiologies of HCC were hepatitis-related: 259 (60.4%) cases of HCV, 31 (7.3%) cases of HBV, and two instances of both. Median maximum tumor diameter was 15.0 (interquartile range, 10.0–21.0) mm. There were 86 tumors in areas of the liver where MTA is difficult. The most common area was near the primary and secondary branches of the intrahepatic portal vein (26 nodules). The cumulative local tumor recurrence rates at 1, 2, and 3 years were 4.4%, 8.0%, and 8.5%, respectively. The cumulative local tumor recurrence rate differed significantly by tumor size group: 6.6%, 13.8%, and 29.4% at three years in the ≤20 mm group (*n* = 483), 20–30 mm group (*n* = 107), and ≥30 mm group (*n* = 17), respectively (*p* < 0.001). The cumulative local tumor recurrence rate was similar despite difficult-to-treat status (*p* = 0.169). In the multivariable analysis, tumor size (>15 mm) (hazard ratio [HR], 2.15; 95% confidence interval [CI], 1.11–4.16; *p* = 0.023) and ablative margin (<3 mm) (HR, 2.94; 95% CI, 1.52–5.71; *p* = 0.001) were significantly associated with local tumor recurrence. Only tumor size (>15 mm) (odds ratio, 3.41 95% CI, 1.53–7.84; *p* = 0.026) was significantly associated with complications. Conclusions: MTA is a safe and effective local ablation therapy for HCC, even for tumors located in areas of the liver where local ablation therapy is difficult.

## 1. Introduction

Hepatocellular carcinoma (HCC) is the leading primary cancer of the liver. It is one of the most common malignancies globally and the cause of substantial health-related problems, making it the third most frequent cause of cancer-related deaths in the world [1]. The Barcelona Clinic Liver Cancer (BCLC) staging system is the most widely used treatment algorithm worldwide [2]. Patients with early-stage HCC based on the BCLC staging system [2] are treated with curative therapies such as surgical resection, transplantation, or locoregional therapies, which include radiofrequency ablation (RFA) and microwave thermosphere ablation (MTA) [3].

Percutaneous RFA has recently become widely used because of its ease of use, safety, and efficacy in the treatment of hepatocellular carcinoma in patients with chronic liver disease [4,5,6,7]. RFA can be performed repeatedly and is particularly useful in reducing intrahepatic recurrence [8].

Microwave ablation (MWA) has been developed as another percutaneous thermal ablation therapy for HCC. Compared to RFA, MWA has the advantage of faster heat generation and higher generated temperatures, making it less susceptible to heat sinking [9]. However, conventional MWAs have several significant disadvantages. First, the ablation zone in conventional MWAs was tear-drop-shaped. Therefore, after developing higher power systems, the risk of thermal damage to subcutaneous tissue and skin was high when performing ablation on subcapsular tumors in the liver with conventional MWA [9]. Second, changes in the surrounding tissue cannot predict the size of the ablation area in conventional MWA [9]. To overcome these disadvantages, Emprint™ (Covidien, Boulder, CO, USA) was developed as a next-generation MWA system using MTA technology. MTA can make predictable spherical ablation zones by incorporating field control, thermal control, and wavelength control technologies into the system. This new system was approved for use in the United States in April 2014 and in Japan in July 2017. In this study, we investigated the clinical outcomes of patients with HCC who underwent next-generation MTA at our hospital.

## 2. Materials and Methods

### 2.1. Patients

All procedures in this retrospective analysis of database records complied with the Declaration of Helsinki. The study protocol was approved by the institutional ethics committee of the Japanese Red Cross Society Himeji Hospital (IRB No. H30-34) based on the Guidelines for Clinical Research issued by the Ministry of Health, Labour and Welfare of Japan. Informed consent to analyze the data was obtained from all patients.

**Figure 1:**
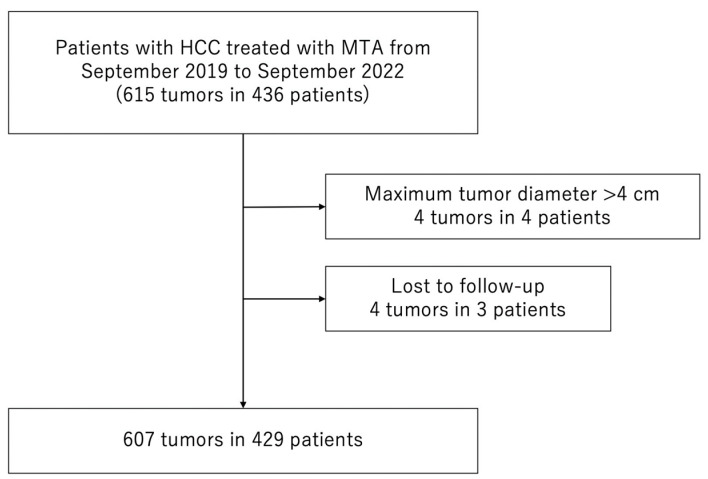
Flow chart of patient selection. MTA, microwave thermosphere ablation; HCC, hepatocellular carcinoma.

Between September 2019 and September 2022, 436 consecutive patients with 615 HCCs were treated with MTA at the Japanese Red Cross Society Himeji Hospital. We excluded patients who met the following criteria: (1) maximum tumor diameter > 40 mm (4 patients with 4 HCCs) or (2) lost to follow-up (3 patients with 4 HCCs). Consequently, 429 patients with 607 HCCs were enrolled in the study (Figure 1).

In September 2019, MTA therapy began to be used as a percutaneous local ablation therapy for HCC at our hospital. Until September 2019, RFA was used as a percutaneous local ablation therapy for HCC at our hospital. However, after the start of MTA therapy, the number of patients undergoing RFA decreased. Since January 2020, MTA therapy has been performed on all patients with HCC for whom percutaneous local ablation therapy was indicated.

HCC etiology was defined as hepatitis B virus in patients positive for hepatitis B virus surface antigen. It was defined as hepatitis C virus in those positive for hepatitis C virus antibodies.

The start of follow-up was defined as when the first MTA treatment was administered. The end of follow-up was defined as the date of the last visit for patients who were alive and the date of death for patients who died during the follow-up period.

### 2.2. Diagnosis and Treatment of HCC

HCC was diagnosed based on the results of multi-phasic contrast-enhanced computed tomography (CECT), gadolinium ethoxybenzyl diethylenetriamine pentaacetic acid (Gd-EOB-DTPA)-enhanced magnetic resonance imaging (MRI), contrast-enhanced ultrasonography (CEUS), pathological examination, or a combination of these modalities, as well as increases in levels of tumor markers such as α-fetoprotein (AFP), des-γ-carboxy prothrombin (DCP), and lens culinaris agglutinin-reactive α-fetoprotein (AFP-L3). The diagnostic criteria of HCC, according to imaging modalities, were based on previous reports of hyper-attenuation at the hepatic arterial phase, hypo-attenuation at the portal venous phase in triple-phase CT or MR imaging, or hyper-enhancement in the arterial phase, hypo-enhancement in the portal venous, and late phases in contrast-enhanced US (CEUS) [10,11].

The Japanese practice guidelines for HCC [12,13] state that MTA is indicated for patients with 3 or fewer HCCs (none > 3 cm). For patients classified as having more severe HCC, MTA was selected based on discussions among hepatologists, surgeons, and radiologists with consideration of the patient’s background such as Eastern Cooperative Oncology Group Performance Status (ECOG-PS), hepatic function, and tolerance for surgery. Informed consent was obtained from each patient.

### 2.3. MTA Technique

The Emprint™ ablation system with a 13-gauge standard antenna (20-cm long) was used for MTA. Prior to percutaneous MTA therapy, 15 mg of pentazocine hydrochloride and 25 mg of hydroxyzine hydrochloride were administered intravenously. Local anesthesia was induced with 5 mL of 1% lidocaine injected through the skin into the peritoneum along a predetermined puncture line. MTA antenna puncture was performed under ultrasound guidance. When the tumor was difficult to visualize using B-mode ultrasound, CEUS or fusion imaging with CECT or Gd-EOB-DTPA-enhanced MRI were used as complementary methods for MTA. B-mode ultrasound and CEUS images were obtained using the Aplio^TM^ i800 system with an 8 MHz convex transducer PVI-482BX (Canon Medical Systems, Otawara, Japan). Artificial pleural effusion or ascites were prepared using 5% glucose solution if needed. When tumor diameter was less than 1.5 cm, output power was initially set at 60 W and changed to 75 W after 1 min. In tumors with a diameter greater than 1.5 cm, starting output power was set at 60 W, changed to 75 W after 1 min, and then changed to 100 W after another minute.

MTA therapy was considered complete when a transient high echogenic region completely covered the target tumor. If coverage of the transient hyperechoic zone was insufficient for the target tumor, additional punctures and ablation were performed in the same treatment session. Needle track ablation at 75 W was performed to prevent hemorrhages. After the antenna was removed from the liver, the needle site was observed using color Doppler ultrasonography. If bleeding from the needle site increased rapidly, a new percutaneous puncture was used to re-ablate the liver surface around the needle site with an MTA antenna to stop the bleeding.

In this study, we defined the following areas of the liver as those where MTA therapy is difficult to perform: caudate lobe and areas near the primary and secondary branches of the intrahepatic portal vein, inferior vena cava, gallbladder, heart, duodenum, abdominal esophagus, collateral veins around the liver, and spleen. We consider that the lesion near the colon is not difficult to treat because the colon can be easily moved away from the cauterization zone by injecting artificial ascites. We defined tumors in contact with the hepatic capsule and raised on the liver surface as protruding from the liver surface.

### 2.4. Evaluation of Treatment Efficacy

Treatment efficacy was evaluated with CECT or MRI at 1–2 days after MTA therapy. Complete ablation was defined as no tumor enhancement with a safety margin ≥ 5 mm on CECT or a post-ablation zone that included the entire target tumor with a safety margin ≥ 5 mm on MRI.

### 2.5. Surveillance for HCC Recurrence after MTA Therapy

Follow-up consisted of regular blood tests and the monitoring of tumor markers such as α-fetoprotein, des-γ-carboxy prothrombin, and lens culinaris agglutinin-reactive α-fetoprotein every 3 months. Multi-phasic CECT, Gd-EOB-DTPA–enhanced MRI, or both were performed every 3–6 months after HCC treatment. When HCC recurrence or disease progression was detected based on radiologic findings, the most appropriate therapy was initiated in each patient based on the Japanese practice guidelines for HCC [12,13].

### 2.6. Evaluation of Outcomes after MTA Therapy

The primary endpoint of this study was local tumor recurrence, which represents treatment failure. Local tumor recurrence was defined as tumor growth that touched the inside or outside of the post-ablation zone.

### 2.7. Safety Evaluation

To evaluate the safety of MTA therapy, the profile and incidence of complications were investigated. Major complications were defined as events leading to substantial morbidity or disability, a higher level of care, hospital admission, or a substantially extended hospital stay [14].

### 2.8. Statistical Analysis

Continuous variables are expressed as medians (interquartile range). The Chi-square test with Fisher’s exact test was used for categorical variables. Actuarial analysis of cumulative local tumor progression was performed using the Kaplan–Meier method; differences were evaluated using the log-rank test. Univariable and multivariable Cox proportional hazards models were used to analyze local tumor progression. In addition, univariable and multivariable regression were used to evaluate the relationship between clinical factors and complications. In this study, the number of events was small and only factors that were significant in the univariable analysis were included as covariates in the multivariable analysis.

Statistical significance was defined as *p* < 0.05. Statistical analysis was performed using JMP^®^ (SAS Institute, Cary, NC, USA).

## 3. Results

### 3.1. Patient Characteristics

The characteristics of the 429 patients at admission are shown in Table 1. There were 293 (68.3%) males and 136 (31.7%) females, with a median age of 76.0 (71.0–82.0) years. The primary etiologies of HCC were hepatitis-related: 259 (60.4%) cases of HCV, 31 (7.3%) cases of HBV, and two instances of both. There were 120 patients (28.0%) who had platelet counts below 100 × 10^3^/µL on admission. The median maximum tumor diameter was 15.0 (10.0–21.0) mm. There were 483 (79.6%) HCCs with maximum tumor diameter of ≤20 mm, 107 (17.6%) with maximum diameter of 20–30 mm, and 17 (2.8%) with maximum diameter of 30–40 mm. There were 304 (70.9%) patients with one tumor, 81 (18.9%) patients with two tumors, 24 (5.6%) patients with three tumors, and 20 (4.7%) patients with ≥ four tumors.

Table 1 includes patients who received multiple treatments during the study period; 304 unique patients were treated with MTA. The characteristics of these 304 patients are shown in Table 2.

There were 201 (66.1%) males and 103 (33.9%) females, with a median age of 73.0 (71.0–81.0) years. The median follow-up period was 23.0 (15.3–30.7) months.

Table 3 shows the number of tumors in areas where MTA is difficult (*n* = 86, including duplications). The most common difficult-to-treat nodules were located near the primary and secondary branches of the intrahepatic portal vein (26 nodules). There were 598 (98.5%) nodules that needed one ablation session, eight (1.3%) that needed two sessions, and one (0.2%) nodule that needed three sessions.

Complete ablation was achieved in 98.7% (599/607) of nodules.

### 3.2. Local Tumor Recurrence

**Figure 2:**
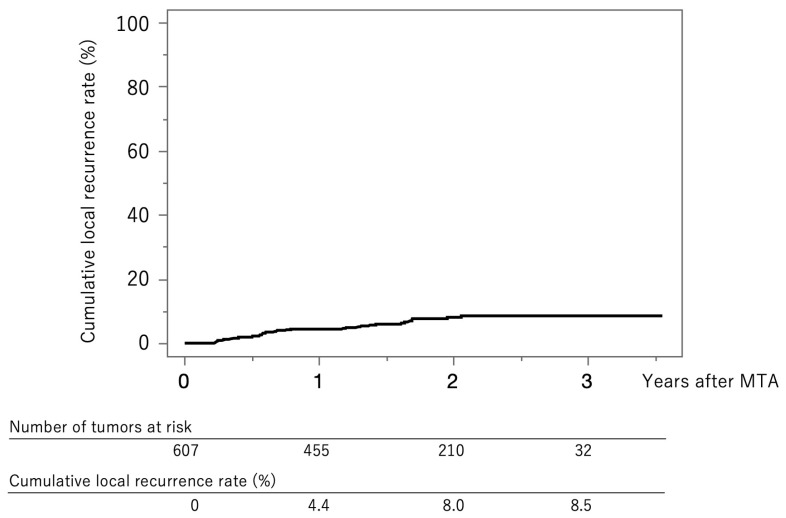
Curve for local tumor recurrence. The cumulative local tumor recurrence rates at 1, 2, and 3 years were 4.4%, 8.0%, and 8.5%, respectively.

Figure 2 shows the curve for local tumor recurrence in this cohort. The cumulative local tumor recurrence rates were 4.4% at 1 year, 8.0% at 2 years, and 8.5% at 3 years.

**Figure 3:**
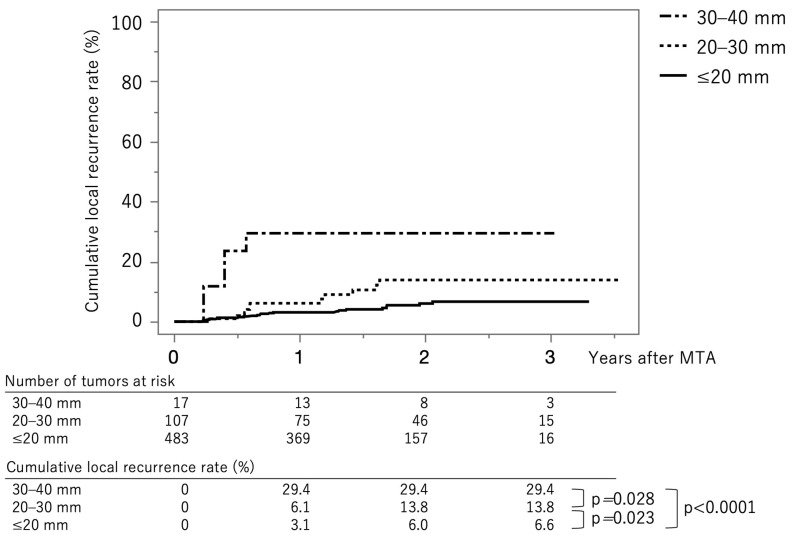
Curves for local tumor recurrence stratified by tumor size group. The cumulative local tumor recurrence rates at 1, 2, and 3 months are 3.1%, 6.0%, and 6.6%, respectively, in the tumor size ≤ 20 mm group (solid line). They were 6.1%, 13.8%, and 13.8%, respectively, in the tumor size 20–30 mm group (dotted line) and 29.4%, 29.4%, and 29.4%, respectively, in the tumor size ≥ 30 mm group (dash-dot-dash line).

Figure 3 shows the curves for local tumor recurrence stratified by tumor size group. The cumulative local tumor recurrence rates at 1, 2, and 3 years were 3.1%, 6.0%, and 6.6%, respectively, in the tumor size ≤ 20 mm group (solid line). The rates were 6.1%, 13.8%, and 13.8%, respectively, in the tumor size 20–30 mm group (dotted line) and 29.4%, 29.4%, and 29.4%, respectively, in the tumor size ≥ 30 mm group (dash-dot-dash line). The *p*-values between ≤20 mm vs. 20–30 mm, 20–30 mm vs. 30–40 mm, and ≤20 mm vs. 30–40 mm were 0.023, 0.028, and <0.0001, respectively (log-rank test). The cumulative local recurrence rates at three years, stratified by tumor size ≤ 15 mm vs. 15–40 mm, were 5.8% and 13.0% (*p* = 0.0029) (Supplementary Figure S1). The cumulative local recurrence rate at three years stratified by the tumor multiplicity; solitary vs. multiple tumors were 6.4% and 10.5% (*p* = 0.191) (Supplementary Figure S2).

**Figure 4:**
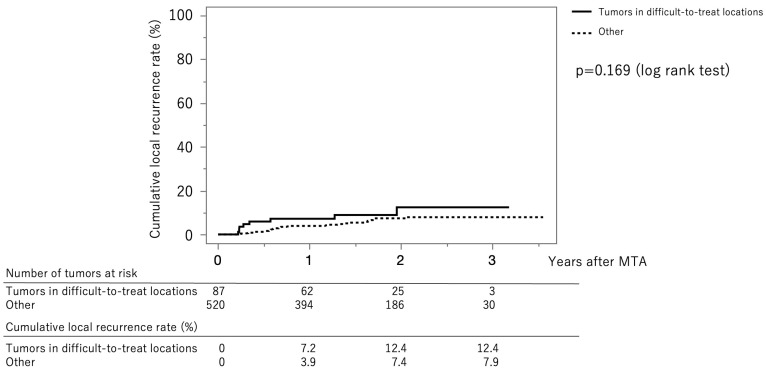
Curves for local tumor recurrence stratified difficult-to-treat status. The cumulative local tumor recurrence rates at 1, 2, and 3 months were 7.2%, 12.4%, and 12.4%, respectively, in the difficult-to-treat group (solid line) and 3.9%, 7.4%, and 7.9%, respectively, in the non-difficult-to-treat group (dotted line) (*p* = 0.169, log-rank test).

Figure 4 shows the curves for local tumor recurrence stratified by difficult-to-treat status. The cumulative local tumor recurrence rates at 1, 2, and 3 years were 7.2%, 12.4%, and 12.4%, respectively, in the difficult-to-treat group (solid line) and 3.9%, 7.4%, and 7.9%, respectively, in the non–difficult-to-treat group (dotted line) (*p* = 0.169, log-rank test).

### 3.3. Clinical Factors Associated with Local Tumor Recurrence

Table 4 shows the univariable and multivariable Cox proportional hazards models associated with local tumor recurrence. Tumor size (>15 mm) (hazard ratio [HR], 2.15; 95% confidence interval [CI], 1.11–4.16; *p* = 0.023) and ablative margin (<3 mm) (HR, 2.94; 95% CI, 1.52–5.71; *p* = 0.001) were significantly associated with local tumor recurrence.

### 3.4. Extrahepatic Metastasis

Recurrence of extrahepatic metastasis was observed in three cases, and the sites of metastasis were lymph node in one case, lung in one case, and bone in one case.

### 3.5. Safety

Table 5 shows the complications in patients overall and stratified by tumor size group. The overall complication rate differed significantly across tumor size groups (*p* < 0.001).

Table 6 shows the univariable, and multivariable regression results for factors associated with complications. Tumor size (>15 mm) (odds ratio, 3.41; 95% CI, 1.53–7.84; *p* = 0.0026) was significantly associated with complications.

## 4. Discussion

In this study, we clarified the clinical outcomes of next-generation MTA in patients with HCC. Although this study only included patients with HCC who received MTA therapy at a single center in Japan, it included over 400 patients with 600 HCCs. This study showed that the cumulative local tumor recurrence rates at 1 and 3 years were 4.4% and 8.5%, respectively. In this study, there were 86 tumors in areas that were difficult to treat with MTA therapy; approximately 30% of those were located near the primary and secondary branches of the intrahepatic portal vein. However, this study did not identify any significant differences in local tumor recurrence by difficult-to-treat status. In the multivariable analysis, tumor size was significantly associated with local tumor recurrence. In fact, the cumulative recurrence rate was significantly higher in the group with large tumor size. In addition, the ablative margin was inversely associated with local tumor recurrence. In our study, 68% of patients had HBV or HCV infection, but as shown in Table 4, HCV or HBV infection was not a significant factor associated with the local recurrence rate.

We used contrast-enhanced CT, MRI, and US of the abdomen, as well as measurement of tumor markers, for the diagnosis of recurrence surveillance. While imaging alone is sufficient to determine intrahepatic recurrence, the presence of elevated tumor markers further strengthens the evidence for recurrence. Even if there is no intrahepatic recurrence, elevated tumor markers may provide proof of suspected extrahepatic metastasis, such as to the chest or bones, and thus are considered necessary. In the present study, three patients had extrahepatic recurrence; one with bone metastasis showed elevated DCP, one with lymph node metastasis showed elevated AFP and AFP-L3, and one with lung metastasis tended toward elevated L3 (Supplementary Table S1). Regarding complications, only tumor size was significantly associated with complications, whereas difficult-to-treat status was not. These results suggest that MTA therapy for HCC located in areas where MTA is difficult is not associated with local tumor progression or post-procedure complications.

In an analysis of 513 patients with 630 HCCs (≤3 cm) who underwent percutaneous RFA (174 patients, 214 HCCs) or MTA (339 patients, 416 HCCs), Tamai et al. reported a significant difference in 3-year local tumor progression rates between the RFA (22%) and MTA (8%) groups (*p* < 0.001) [15]. They found that ablation procedure (MTA; HR, 0.565; 95% CI, 0.437–0.731; *p* < 0.001) and tumor diameter (per mm; HR, 1.070; 95% CI, 1.030–1.113; *p* = 0.001) were independent factors associated with local tumor progression in a multivariable analysis [15]. In addition, they found that the total complication rate was significantly lower in the MTA group (8.0%, 26/339) than in the RFA group (14.0%, 25/174) (*p* < 0.05), particularly for bile duct injury (3.0% (11/339) versus 9.0% (15/174); *p* = 0.010). In this study, we found that the cumulative local tumor recurrence rate at 3 years was 8.5% in our cohort, which included patients with tumors ≥ 3 cm. In this cohort, the total complication rate was 5.4% (33/607). These results suggest that MTA therapy can achieve good outcomes in patients with HCC for whom percutaneous ablation therapy is indicated. The advantage of this study is that our study included more patients and tumors that underwent MTA than the study by Tamai et al.

The Emprint™ Ablation System with Thermosphere™ Technology (Covidien) is an improved version of the Evident™ Microwave Ablation System developed by the same manufacturer. The new device uses a frequency of 2450 MHz and consists of a 100 W generator with a high-efficiency reusable cable and an ablation pump that cools the antenna during ablation. The U.S. Food and Drug Administration approved this system on 28 April 2014, and it was approved for use in Japan on 1 November 2016. This new generation MWA system attempts to address the limitations of conventional systems. It is designed to create a predictable, large, spherical ablation zone that is unaffected by changes in the tissue environment [16]. Thus, predictable resection results and outcomes are obtained regardless of the target site or tissue type [17]. Large, accurate, and predictable spherical ablation zones are maintained throughout the procedure by three different types of controls (field control, thermal control, and wavelength control) [18]. Thermosphere™ technology maintains wavelength control by creating a constant, stable environment around the probe shaft and circulating sterile saline solution along the shaft. This minimizes the change in dielectric constant directly under the probe and maintains a shorter wavelength. It also ensures that the migration pattern of electrons in the probe is maintained, providing reliable field control. However, there have been few studies regarding MTA therapy that have evaluated treatment of HCC in real-world clinical practice. In this study, we clarified the outcomes of this therapy for HCC at a high-volume center in Japan.

The main limitations of this study include its small number of patients and retrospective nature at a single center in Japan. Further prospective studies with more patients from multiple centers are warranted. Another limitation of this study was that the outcomes have not been compared with those of RFA, which had been the mainstay of local ablation therapy for HCC. Further prospective studies that compare clinical outcomes of MTA and RFA in the real-world clinical setting should be performed.

In conclusion, MTA is a safe and effective local ablation therapy for HCC, even when the tumor is located in an area of the liver where local ablation therapy is difficult. Further studies are warranted to confirm these findings in other populations.

## Figures and Tables

**Table 1:** Characteristics of patients and HCCs at the time of admission (*n* = 429).

Characteristics	
Age at admission (years)	76.0 (71.0–82.0)
Gender (male/female) *	293/136
HCC etiology (hepatitis B virus/hepatitis C virus/B + C/non-B, non-C)	31/259/2/137
Child-Pugh classification (A/B)	379/50
ECOG-PS (0/1/2)	384/41/4
Maximum tumor diameter (mm) *	15.0 (10.0–21.0)
Number of tumors (1/2/3/≥4)	304/81/24/20
Number of prior treatments for HCC (0/1/≥2)	120/99/210
α-fetoprotein (ng/mL) *	6.1 (3.3–13.4)
*Lens culinaris* agglutinin-reactive α-fetoprotein (%) *	0.0 (0.0–9.6)
Des-γ-carboxy prothrombin (mAU/mL) *	28.0 (19.0–64.1)

* Data are expressed as medians (interquartile range). ECOG-PS, Eastern Cooperative Oncology Group performance status; HCC, hepatocellular carcinoma.

**Table 2:** Characteristics of unique patients that underwent MTA (*n* = 304).

Characteristics	
Age at first MTA *	76.0 (71.0–81.0)
Gender (male/female) *	201/103
Etiology (hepatitis B virus/hepatitis C virus/B+C/non-B, non-C)	25/176/2/101
Follow-up duration (months) *	23.0 (15.3–30.7)

* Data are expressed as medians (interquartile range). MTA, microwave thermosphere ablation.

**Table 3:** Number of tumors in areas of the liver where MTA is difficult (*n* = 86; including duplications).

Areas of the Liver	Number of Tumors
Primary and secondary branches of intrahepatic portal vein	26
Adjacent to the heart	25
Adjacent to the inferior vena cava	19
Adjacent to the gallbladder	17
Caudate lobe	9
Adjacent to the duodenum	5
Adjacent to the esophagus	1
Adjacent to a collateral vein	1

MTA, microwave thermosphere ablation.

**Table 4:** Factors associated with local tumor recurrence.

Factors	Univariable Analysis			Multivariable Analysis		
	HR	95% CI	*p*	HR	95% CI	*p*
Difficult-to-treat location (yes)	1.72	0.79–3.75	0.170	1.28	0.58–2.84	0.535
Tumor size (>15 mm)	2.59	1.35–4.98	0.004	2.15	1.11–4.16	0.023
Ablative margin (<3 mm)	3.45	1.81–6.57	<0.001	2.94	1.52–5.71	0.001
Protruding from the liver surface (yes)	1.17	0.58–2.36	0.660			
Multiple tumors (yes)	0.65	0.34–1.25	0.190			
Prior HCC treatment (yes)	0.85	0.42–1.71	0.640			
α-fetoprotein (>10 ng/mL)	1.42	0.74–2.70	0.290			
*Lens culinaris* agglutinin-reactive α-fetoprotein (>10%)	1.58	0.62–4.02	0.335			
Des-γ-carboxy prothrombin (>28 mAU/mL)	0.56	0.29–1.07	0.080			
HBV vs. non-virus	0.83	0.18–3.76	0.814			
HCV vs. non-virus	1.32	0.65–2.68	0.447			
ALT (>40 IU/L)	1.02	0.43–2.44	0.970			
Platelet (<100 × 10^3^/μL)	0.56	0.25–1.28	0.168			

CI, confidence interval; HCC, hepatocellular carcinoma, HR, hazard ratio.

**Table 5:** Complications due to MTA.

Complication (Including Duplications)	Total (*n* = 607)	Tumor Size Group		
		≤20 mm	20–30 mm	30–40 mm
Any	33 (5.4%)	17 (3.5%)	11 (10.3%)	5 (29.4%)
Portal vein thrombus	9 (1.5%)	5 (1.0%)	4 (3.7%)	0 (0.0%)
Biloma	9 (1.5%)	5 (1.0%)	2 (1.9%)	2 (11.8%)
Bile duct dilatation	6 (1.0%)	3 (0.6%)	2 (1.9%)	1 (5.9%)
Pleural effusion	4 (0.7%)	3 (0.6%)	0 (0.0%)	1 (5.9%)
Hemorrhage	2 (0.3%)	1 (0.2%)	1 (0.9%)	0 (0.0%)
Hepatic infarction	1 (0.2%)	1 (0.2%)	0 (0.0%)	0 (0.0%)
Pneumothorax	2 (0.3%)	0 (0.0%)	2 (1.9%)	0 (0.0%)
Heat injury of the diaphragm	1 (0.2%)	1 (0.2%)	0 (0.0%)	0 (0.0%)
Heat injury of the right kidney	1 (0.2%)	0 (0.0%)	0 (0.0%)	1 (5.9%)

MTA, microwave thermosphere ablation.

**Table 6:** Factors associated with complications.

Factors	Univariable Analysis			Multivariable Analysis		
	Odds Ratio	95% CI	*p*	Odds Ratio	95% CI	*p*
Difficult-to-treat location (yes)	2.38	1.02–5.16	0.0460	2.01	0.83–4.48	0.116
Total ablation time (>5 min)	2.53	1.11–5.36	0.0278	0.90	0.29–2.48	0.740
Tumor size (>15 mm)	3.68	1.79–8.03	0.0004	3.41	1.53–7.84	0.026
Number of punctures (≥2)	3.50	1.34–8.17	0.0129	2.51	0.77–7.97	0.126
Ablative margin (≥3 mm)	0.53	0.26–1.10	0.0873			
Protruding from the liver surface (yes)	1.66	0.77–3.41	0.190			
Multiple tumors (yes)	0.72	0.35–1.46	0.360			
Prior HCC treatment (yes)	0.55	0.27–1.18	0.120			
Child-Pugh classification (B)	0.87	0.53–1.29	0.500			

CI, confidence interval; HCC, hepatocellular carcinoma.

## Data Availability

The datasets are available from the corresponding author upon reasonable request.

## Supplementary Materials

The following supporting information can be downloaded at: https://www.mdpi.com/article/10.3390/jcm12247577/s1, Figure S1. Curves for local tumor recurrence stratified by tumor size group. The cumulative local recurrence rate at three years stratified by tumor size ≤ 15 mm vs. 15–40 mm were 5.8% and 13.0% (*p* = 0.0029). Figure S2. Curves for local tumor recurrence stratified by the tumor multiplicity. The cumulative local recurrence rate at three years stratified by the tumor multiplicity; solitary vs. multiple tumors were 6.4% and 10.5% (*p* = 0.191). MTA, microwave thermosphere ablation. Table S1. Tumor markers at the occurrence of extrahepatic metastasis.

## Abbreviations

HCC	hepatocellular carcinoma
BCLC	Barcelona Clinic Liver Cancer
RFA	radiofrequency ablation
MTA	microwave thermosphere ablation
MWA	microwave ablation
AFP	α-fetoprotein
AFP-L3	Lens culinaris agglutinin-reactive α-fetoprotein
DCP	Des-γ-carboxy prothrombin
CECT	contrast-enhanced computed tomography
Gd-EOB-DTPA	gadolinium ethoxybenzyl diethylenetriamine pentaacetic acid
MRI	magnetic resonance imaging
CEUS	contrast-enhanced ultrasonography
ECOG-PS	Eastern Cooperative Oncology Group performance status
HR	hazard ratio
CI	confidence interval
