# Supplementary material for: Clinical Outcomes of Next-Generation Microwave Thermosphere Ablation for Hepatocellular Carcinoma with Primarily Hepatitis-Related Etiology
Source: J Clin Med. 2023 Dec 8;12(24):7577. doi: 10.3390/jcm12247577 (PMC10743866; doi:10.3390/jcm12247577)
Supplement: Supplementary file 1 [file jcm-12-07577-s001.zip › jcm-2707214-supplementary.pdf]

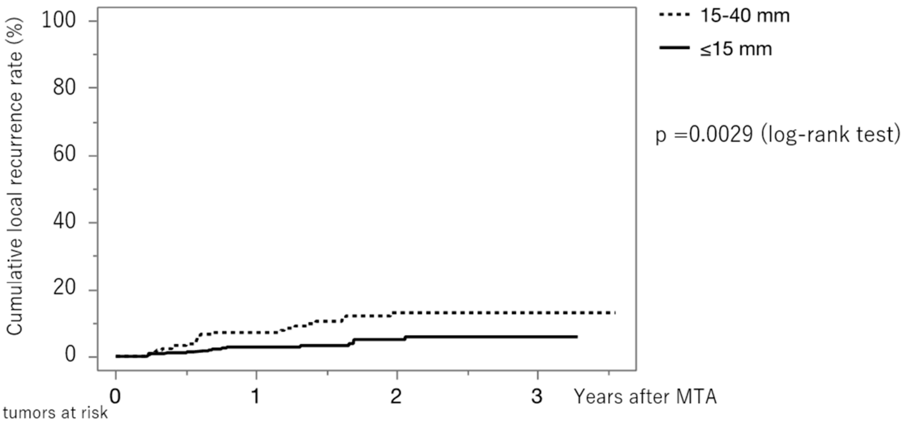

Number of tumors at risk				
15–40 mm	224	163	90	19
≤15 mm	383	293	120	14

Cumulative local recurrence rate (%)				
15–40 mm	0	7.1	13.0	13.0
≤15 mm	0	2.8	5.0	5.8

Supplemental Figure S1.

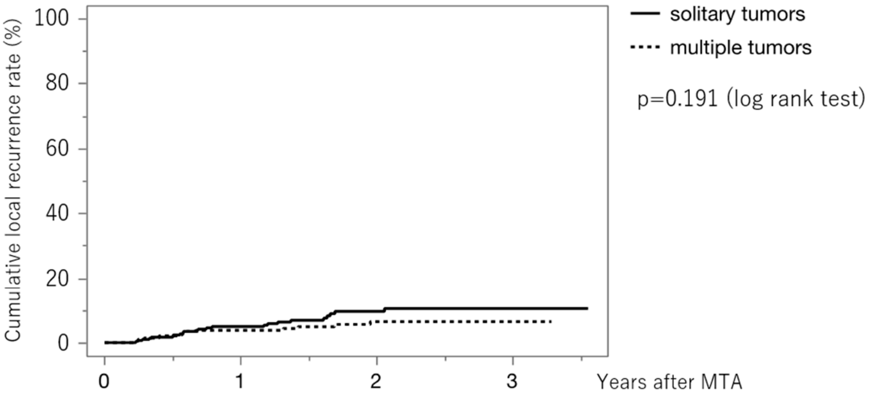

Number of tumors at risk				
Solitary tumor	303	232	106	22
Multiple tumors	304	225	105	12

Cumulative local recurrence rate (%)				
Solitary tumor	0	4.9	9.6	10.5
Multiple tumors	0	3.8	6.4	6.4

Supplemental Figure S2.

Supplementary Table S1. Tumor markers at the occurrence of extrahepatic metastasis

	Case 1.	Case 2.	Case 3.
Lesion of extrahepatic metastasis	bone	lymph node	lung
α-fetoprotein (ng/mL) *	1.5	47.7	9.3
Lens culinaris agglutinin-reactive α-fetoprotein (%) *	<0.5	13.3	7.8
Des-γ-carboxy prothrombin (mAU/mL) *	207.0	14.0	20.0
